# Multi-Junction Solar Module and Supercapacitor Self-Powering Miniaturized Environmental Wireless Sensor Nodes

**DOI:** 10.3390/s24196340

**Published:** 2024-09-30

**Authors:** Mara Bruzzi, Giovanni Pampaloni, Irene Cappelli, Ada Fort, Maurizio Laschi, Valerio Vignoli, Dario Vangi

**Affiliations:** 1 Department of Physics and Astronomy, University of Florence, Via G. Sansone 1, 50019 Florence, Italy; giovanni.pampaloni@stud.unifi.it; 2 National Institute of Nuclear Physics INFN, Florence Section, Via G. Sansone 1, 50019 Florence, Italy; 3 Department of Information Engineering and Mathematics, University of Siena, via Roma 56, 53100 Siena, Italy; cappelli@diism.unisi.it (I.C.); ada.fort@unisi.it (A.F.); valerio.vignoli@unisi.it (V.V.); 4 Department of Industrial Engineering, University of Florence, Via S. Marta 3, 50039 Florence, Italy; maurizio.laschi@unifi.it (M.L.); dario.vangi@unifi.it (D.V.)

**Keywords:** multi-junction photovoltaic module, supercapacitors, wireless sensor nodes, CO_2_ sensors, self-powered sensors, environmental gas monitoring

## Abstract

A novel prototype based on the combination of a multi-junction, high-efficiency photovoltaic (PV) module and a supercapacitor (SC) able to self-power a wireless sensor node (WSN) for outdoor air quality monitoring has been developed and tested. A PV module with about an 8 cm^2^ active area made of eight GaAs-based triple-junction solar cells with a nominal 29% efficiency was assembled and characterized under terrestrial clear-sky conditions. Energy is stored in a 4000 F/4.2 V supercapacitor with high energy capacity and a virtually infinite lifetime (10^4^ cycles). The node power consumption was tailored to the typical power consumption of miniaturized, low-consumption NDIR CO_2_ sensors relying on an LED as the IR source. The charge/discharge cycles of the supercapacitor connected to the triple-junction PV module were measured under illumination with a Sun Simulator device at selected radiation intensities and different node duty cycles. Tests of the miniaturized prototype in different illumination conditions outdoors were carried out. A model was developed from the test outcomes to predict the maximum number of sensor samplings and data transmissions tolerated by the node, thus optimizing the WSN operating conditions to ensure its self-powering for years of outdoor deployment. The results show the self-powering ability of the WSN node over different insolation periods throughout the year, demonstrating its operation for a virtually unlimited lifetime without the need for battery substitution.

## 1. Introduction

Mitigation and adaptation to global warming and climate change represent a priority strategy for sustainable economic growth and development [1,2]. Air quality control through global and local monitoring of toxic gases is a powerful tool in view of achieving this final objective. Recent research has been directed toward the development of extended wireless networks of miniaturized and energy-sustainable sensors [3,4,5] composed of power-efficient storage elements and small-sized nodes. Wireless sensor nodes (WSNs) are, in general, equipped with an energy-harvesting and storage section consisting of a photovoltaic (PV) module, battery storage and a power management system (PMS) [6]. The amount of energy/power managed by these systems is mainly affected by the embedded sensors and the data transmission tasks. For gas monitoring tasks, optical-based carbon dioxide (CO_2_) sensors, chemoresistive sensors, particulate sensors and catalytic sensors are among the most expensive in terms of energy demand [7,8,9,10]. This makes them virtually incompatible with ultra-low-power applications relying on harvesting sources. Indeed, the most popular and accurate CO_2_ gas sensors exploit Non-Dispersive InfraRed (NDIR) technology [7] based on an IR source, traditionally an incandescent lamp, which may have a typical power consumption of up to 300 mW [8]. Recently, IR LED sources working in pulse mode have become available, with power consumption reduced to about 10 mW [7]. Chemiresistive sensors, due to their high-temperature operation, have a power consumption of the same order of magnitude [9], while particulate sensors may require even one order of magnitude higher [10].

To manage such power consumption, traditional PV modules based on silicon, characterized by a 10–20% efficiency depending on its crystalline quality [11], require a non-negligible size. Miniaturized devices can be obtained by means of next-generation PV modules characterized by considerably higher energy conversion efficiencies. Consolidated prototypes based on GaAs-based triple-junction solar cells, in fact, may achieve 30% efficiency under terrestrial Sun irradiation [12], allowing the PV module’s effective area exposed to the Sun to be almost halved with respect to silicon-based devices.

A further advance in the node energy-harvesting architecture may concern the energy storage aspect, increasing the device’s eco-friendliness by excluding Li batteries, which have a non-negligible environmental impact due to their limited lifetime, flammability and toxicity. Supercapacitors recently emerged as a valid alternative to traditional batteries due to their advantages in terms of fast charging, high power density, long life cycle and wide temperature range of operation [13,14]. Their potential for high-performance self-powered wireless multi-sensing microsystems has been recently exploited [15]. In fact, a WSN power supply system with a 35 cm^2^ photovoltaic surface coupled with supercapacitors (2 × 25 F capacitance and 2.7 V voltage) for permanent operation was presented and discussed in [15].

The activity proposed in this paper represents a significant improvement of this previous pioneering work by making use of a hybrid supercapacitor, characterized by higher capacitance, power and energy density [16,17,18], in conjunction with a high-efficiency triple-junction PV module in place of the more common polycrystalline or monocrystalline silicon PV cells. These devices, once accurately characterized with laboratory instrumentation, were integrated into a small-size WSN to prove their permanent outdoor operation. The WSN is designed to have an average power demand of about 10 mW, which is the typical consumption of solid-state LED-based sensing devices for CO_2_ monitoring [7]. However, the obtained results can be generalized to various sensing devices for environmental and air quality monitoring, such as temperature and humidity sensors or electrochemical sensors for O_2_, CO, NO_x_ and Volatile Organic Compound (VOC) measurements, which are characterized by power requirements similar to or lower than those experienced in the presented tests.

The system architecture and the PV module and supercapacitor characterization are presented in Section 2 (Materials and Methods). Section 3 describes the main experimental results obtained with the whole prototype, both in the laboratory with a Sun Simulator lamp and outdoors. Section 4 presents the model developed from the test outcomes to optimize the WSN operating conditions in view of ensuring the virtually unlimited self-powering of the prototype. Finally, the conclusions and outlooks of the presented research are reported in Section 5.

## 2. Materials and Methods

**Figure 1:**
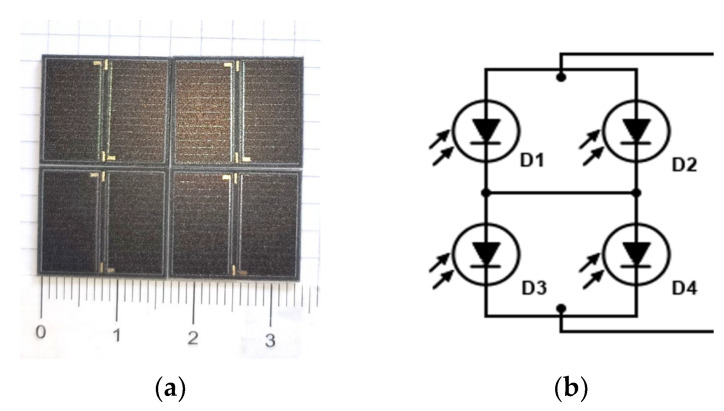

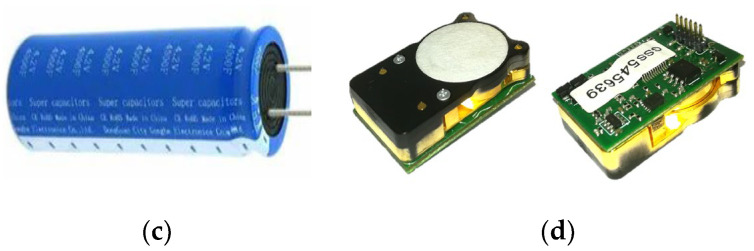
The PV module composed of eight triple-junction solar cells: (**a**) photograph and (**b**) circuit schematics (each diode D is composed of two triple-junctions in a series). (**c**) A photo of the hybrid supercapacitor used in this work. (**d**) A picture of back and front views of the CO_2_ sensor emulated in this work.

We prepared the prototype PV module starting from four GaAs-based solar cells (MSCM-4.5-14.8-30%) produced by Shanghai YIM Machinery Equipment Co., Ltd. (Shanghai, China) [19]. Each solar cell is composed of a series of two triple junctions. They are dust- and waterproof in accordance with the IP67 protocol and guaranteed under (−80 °C, +80 °C) temperature conditions for 8 years of operation. The area of each cell is 17 × 16.4 mm^2^, and its weight is 0.7 g; the cell active area is 2.1 cm^2^. The nominal operating voltage and current are 4.5 V and 14.8 mA, respectively, and the efficiency is η = 29% at null Air Mass (AM0) and T = 25 °C, namely, standard conditions for characterizing photovoltaic modules for extraterrestrial applications. A photograph and the circuit schematics of the PV module we assembled as a series of two solar cells in parallel are shown in Figure 1a,b. The operating current and voltage values expected in this configuration from nominal specifications are 29.6 mA and 9 V, corresponding to 266 mW peak power.

Table 1 shows the nominal specifications of the supercapacitor (C424000R) used in this study, manufactured by DongGuan GongHe Electronics Co., Ltd. (Dongguan, Guangdong, China), with a cylindrical shape, a 69 mm height, and a 24 mm diameter, characterized by the protection class IP30 and 70 g weight [18]. A photograph of the hybrid supercapacitor used in this work is reported in Figure 1c. 

Finally, the sensor emulated in the following tests is the CozIR-LP by Gas Sensing Solutions Ltd. (Cumbernauld, Glasgow, UK) [7], whose back and front pictures are presented in Figure 1d. It is an NDIR sensor for CO_2_ measurements (30 ppm of resolution) based on a solid-state LED, which dissipates less energy than other sensing solutions based on filament lamps. The low power consumption of the CozIR-LP and its supply voltage range make it compatible with battery-based operations, allowing it to be used in a wide range of Internet of Things applications. Moreover, communication through the UART interface makes it extremely versatile and easily integrable with several types of MCU platforms.

The used power management system (PMS) is from Waveshare, Shenzhen, China [20]. It has a 65.2 mm × 56.2 mm size, is compatible with 6 V to 24 V solar panels and supports a 14500 Li-ion battery (850 mAh) or an equivalent rechargeable storage element. The voltage across the storage element must be between 2.9 V and 4.2 V. The system provides a regulated output at 5 V/1 A or 3.3 V/1 A to power up a connected load. The specifications of the PMS are given in Table 2. In this study, we modified the battery case of the PMS to match the supercapacitor dimensions. The node is connected to the 3.3 V/1 A output. To select the maximum power point (MPP), this PMS applies the constant-voltage method, automatically setting the output voltage V_out_ as a fraction of the open-circuit voltage V_oc_ by a voltage divider. By selecting the open-circuit voltage V_oc_ = 9 V within the range of possible values (6–9–12–18–24 V), the actual voltage output fixed by the PMS is V_out_ = 7.65 V = 0.84 V_oc_.

**Figure 2:**
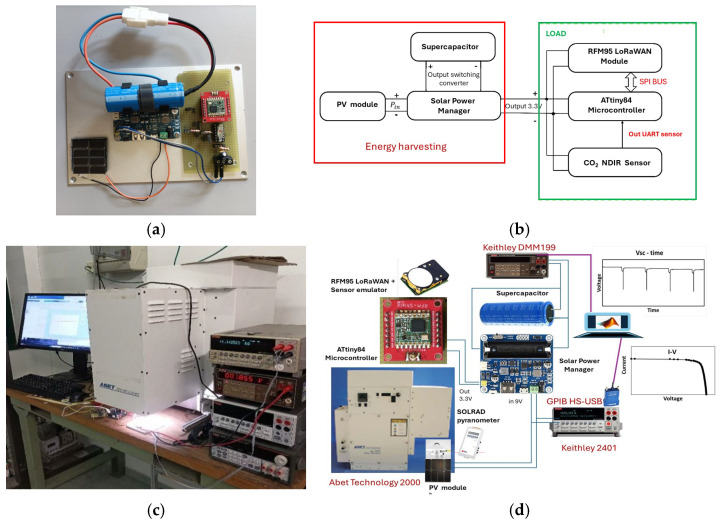
(**a**) A photograph of the system used in this study, composed of the PV module made of four triple-junction solar cells, the PMS carrying the supercapacitor and the wireless sensor node with resistance simulating typical sensor consumption. (**b**) The system flowchart, including the emulated CO_2_ sensor. (**c**) A photograph of the system during a measurement under the Sun Simulator. (**d**) A block diagram of the measurement system.

A picture of the whole system under study, composed of the PV module made of four triple-junction solar cells, the power manager system (PMS) carrying the supercapacitor (SC) and the wireless sensor node (WSN) with a resistive load emulating a sensor, is shown in Figure 2a. The flowchart of the entire system is sketched in Figure 2b.

Measurements were carried out both indoors and outdoors. Laboratory tests were carried out by means of a Sun Simulator 2000 (Abet Technology, Milford, CT, USA) based on a 150 W Xe lamp [21]. The radiation intensity and the temperature of the module were continuously monitored by a Kipp & Zonen (OTT HydroMet B.V., Delft, The Netherlands) CMP3 pyranometer equipped with the SOLRAD read-out system [22] and a Pt1000 thermometer. The CMP3 has a sensitivity S = 28 ± 4 μV/(W/m^2^), a spectral range of 300–2800 nm and a response time of about 1 s. The uncertainty of the intensity measured was evaluated after repeated measurements in the same conditions, up to ten times, resulting in a 5% error. The Qmini WIDE VIS spectrometer (Broadcom Inc., Palo Alto, CA, USA) with a spectral resolution of 1.5 nm and a (225 nm, 1030 nm) measurement range based on a CCD, was used to monitor the radiation spectrum [23]. A Keithley (Tektronix, Bracknell, UK) 2401 (source/electrometer was used to measure the PV module current–voltage (I–V) characteristics. The instrument is characterized by a maximum measured voltage V_max_ = 21 V, 100 μV resolution, accuracy ΔV = 0.015%V + 1.5 mV and maximum current supplied I = 105 mA. The uncertainty of the voltage measurement was experimentally determined by repeating measurements in the same operating conditions, up to ten times, yielding values within the instrument accuracy. A picture of the system running when the PV module is exposed to Sun Simulator illumination is shown in Figure 2c. The voltage across the supercapacitor is measured by a Keithley DMM 199, which is able to perform voltage measurements in the range from 300 V to 1 μV with a 5 1⁄2-digit resolution. Data are collected through GPIB/NI protocols by means of the MATLAB R2020b instrument control toolbox. A block diagram of the whole measurement system is shown in Figure 2d.

A prototype sensor node was exploited using a network infrastructure relying on long-range (LoRa) modulation and the associated LoRa Wide-Area Network (LoRaWAN) protocol [24]. In detail, the sensor node transmitted the sampled data to a LoRa gateway according to the “class A” LoRaWAN standard implementation, and then the gateway was in charge of redirecting the packets to the Chirpstack cloud LoRaWAN server, which in turn decoded and decrypted the received packets and sent the retrieved information to an SQL database for storage. With the aim of testing the operation of a general-purpose device, a standard low-power consumption system architecture was designed, integrating a low-power 8-bit AVR ATtiny84 microcontroller by Microchip and an RFM95 LoRa module by HopeRF (Shenzhen, China). The MCU was programmed according to a sleep routine that foresaw the periodic wakeup of the MCU at fixed time intervals.

**Figure 3:**
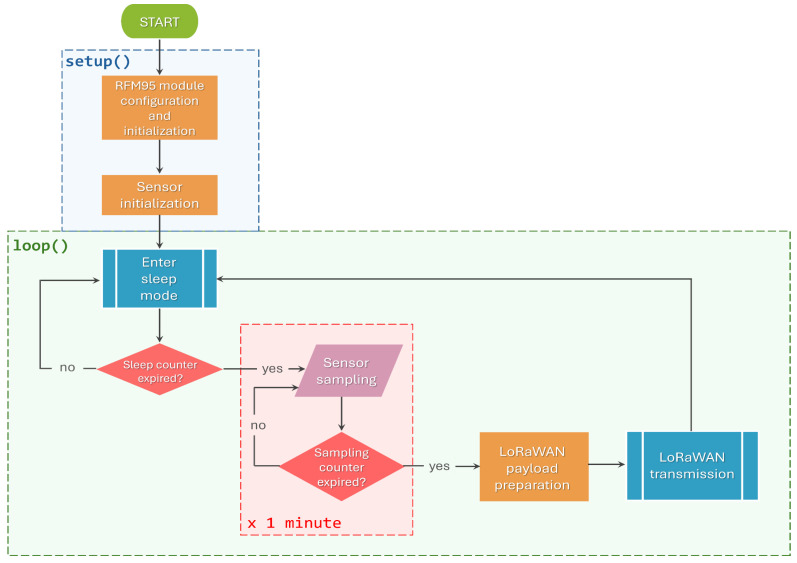
A flowchart describing the code executed by the MCU.

Concerning the radio transmission settings, a transmitting frequency of 868 MHz, output power of 14 dBm, coding rate (CR) of 4/8, spreading factor (SF) of 12 and bandwidth (BW) of 125 kHz were set. These settings were chosen as a tradeoff between the low-consumption requirement and transmission reliability in the case of a hypothetical outdoor application scenario, which may be affected by critical issues related to noise, signal attenuation, reduced radio coverage and long distances between sensor nodes and gateways. Indeed, CR = 4/8 guarantees the best error correction, while SF = 12 provides the highest receiver sensitivity and the lowest packet loss, which are important aspects to be accounted for in deployments of monitoring systems in remote and critical scenarios. Finally, a resistive load was embedded in view of emulating an average power consumption of 10 mW as typical sensor consumption. The node was programmed to wake up for 1 min to simulate its consumption during sensor measurement, and then a radio transmission was performed. The flowchart describing the program executed by the MCU is presented in Figure 3.

### 2.1. PV Module Characterization

**Figure 4:**
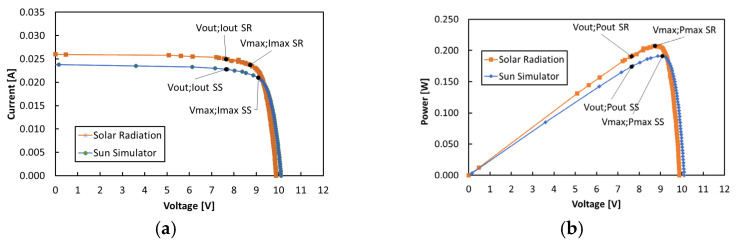
(**a**) I-V and (**b**) P-V characteristics of the triple-junction module under irradiation outdoors with a clear sky and under the Sun Simulator, both under 1000 W/m^2^ intensity and AM1.5G conditions.

The PV module we assembled is a novel prototype with unknown performance under both outdoor terrestrial and indoor laboratory conditions. We measured the current–voltage (I-V) characteristics under 1000 W/m^2^ in AM1.5G conditions (solar radiation, SR) and under Sun Simulator irradiation (SS) at 1000 W/m^2^ intensity and with the AM1.5G filter. The results are shown in Figure 4a,b, respectively, for current–voltage (I-V) and power–voltage (P-V) curves. The maximum power coordinates (V_max_,I_max_) and (V_max_,P_max_) are depicted in both plots, together with the actual output coordinates (V_out_,I_out_) and (V_out_,P_out_), respectively, due to the PMS selection at 85% V_oc_. The relevant photovoltaic parameters obtained from measurements are reported in Table 3. Here, $I_{sc}$ is the short-circuit current, and FF is the fill factor, while η_out_ and η_max_ are the energy conversion efficiencies, respectively, in the output and maximum power point conditions.

**Figure 5:**
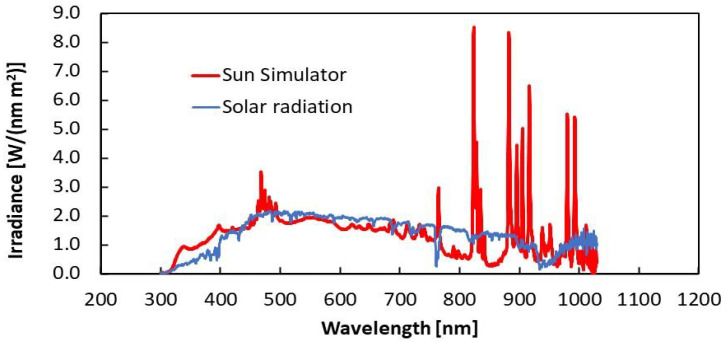
Irradiance from the Sun Simulator compared to the solar radiation spectrum measured with the spectrometer in the case of 1000 W/m^2^ intensity and the AM1.5G configuration.

The measured parameters of the PV module are lower than the nominal ones, probably due to the irradiation conditions being different from nominal extraterrestrial parameters and characterized by a higher temperature. In general, the measurements evidence that the PV module performs better under solar irradiation than under Sun Simulator illumination. This is probably due to a spectrum mismatch of the Sun Simulator with respect to the actual solar radiation spectrum. This is shown in Figure 5, which compares the two spectra measured with the Qmini spectrometer in the range 300–1030 nm for the same intensity (1000 W/m^2^) in AM1.5G conditions. A decrease in efficiency of about 2% is due to the differences between the output current and voltage coordinates (V_out_,I_out_) selected by the MPP algorithm of the PMS and the maximum power point coordinates (V_max_,I_max_).

### 2.2. Supercapacitor Characterization

**Figure 6:**
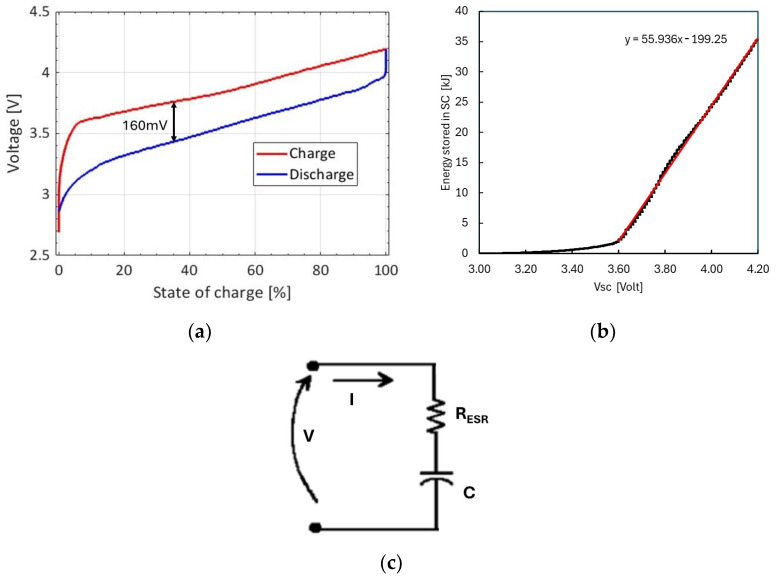
(**a**) Voltage vs. SoC characteristics of the SC measured during a charge/discharge cycle; (**b**) energy stored in the SC as a function of the voltage across the supercapacitor, V_sc_. Red line shows linear best fit of data in the range 3.60–4.2 V. (**c**) the SC equivalent circuit.

**Figure 7:**
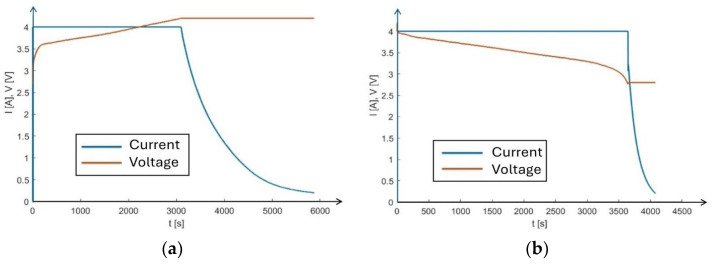
The current supplied and voltage measured as a function of time during the supercapacitor (**a**) charge and (**b**) discharge cycles shown in Figure 6a.

The hybrid supercapacitor is a novel prototype with unknown performance during charge/discharge. An Electro-Automatik EA-PS 9080-60 T (Viersen, Germany) source and Electro-Automatik EA EL 9080-45T (Viersen, Germany) were used to measure the charge/discharge cycles of the supercapacitor in terms of voltage vs. state-of-charge (SoC) characteristics. Charge/discharge cycles expressed in terms of voltage across the supercapacitor as a function of its state of charge (SoC) are shown in Figure 6a. The currents flowing in the SC and voltage across electrodes during charge/discharge cycles are given in Figure 7a,b. The hysteresis effect observed in the charge/discharge cycles of Figure 6a can be explained in terms of equivalent internal resistance R_ESR_ (see Figure 6c), adding a voltage contribution V_R_ = R_ESR_I during measurements. R_ESR_ can be evaluated from the voltage difference between the two linear parts of the charge/discharge curves, ΔV = 2V_R_ = 160 mV, measured at the same current: I = 4 A. We obtain R_ESR_ = 20 mΩ, in agreement with the nominal value (see Table 2). To evaluate the energy stored by the supercapacitor, we note that, considering the capacitance value, C = 4000 F, the highest voltage, 4.2 V, corresponds to a charge Q_max_ = CV_max_ = 16,800 C obtained at SoC = 100%. Then, we can determine the energy stored in the supercapacitor as $U=\frac{1}{2}QV$. Figure 6b shows that the stored energy *U* is linearly dependent on the voltage across the supercapacitor electrodes, V_SC,_ in the range (3.6 V, 4.2 V). Here, a change ΔV_sc_ = 1 mV corresponds to exchanged energy on the supercapacitor U_sc_ = 55.94 J = 15.54 mWh. We note that, for proper operation, the supercapacitor must be kept working within this voltage range.

## 3. Experimental Results

### 3.1. Laboratory Tests 

**Figure 8:**
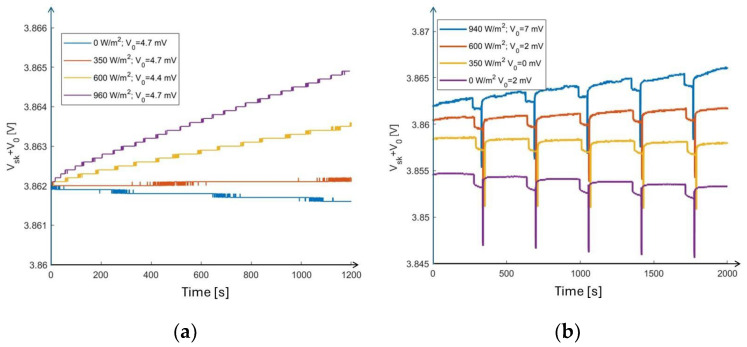
Voltage measured across the SC as a function of time with the PV module under the Sun Simulator at fixed intensities in the case where (**a**) the node is switched off and (**b**) the node is transmitting every 5 min. Curves are shifted by V_0_ to facilitate their comparison.

Laboratory tests of the whole system were carried out by exposing the PV module to illumination with selected intensities from the Sun Simulator. The node was woken for 1 min at programmable time intervals with a power consumption of 10 mW. Meanwhile, the voltage across the SC was measured (at approximately 0.5 s time intervals) by means of the 5½-digit multimeter. Prior to this, the SC had been charged to work in the proper linear voltage range: 3.6 ÷ 4.2 V. The results for light intensities of 0, 350, 600 and 960 W/m^2^ are shown in Figure 8a,b, which depict cases where the node, respectively, is switched off and on, sampling and transmitting every 5 min. The same tests were repeated for transmission every 15 min and 30 min. When the node is switched off, the voltage across the SC changes monotonically, increasing at higher intensities (600 W/m^2^, 960 W/m^2^), remaining almost constant at intermediate intensity (350 W/m^2^) and slightly decreasing in dark conditions. From these measurements, we can determine the consumption of the system during the sleeping periods. In Figure 8b, we observe that each time the node wakes up during the 1-min-long sensing phase, a voltage drop, ΔV = R I, occurs, with I being the current flowing from the PV module and R being the resistive load. The spike at the end of each node operation indicates the data transmission phase.

### 3.2. Outdoor Tests

Outdoor tests of the whole system were carried out in Florence, Italy, at nighttime and in daylight under clear-sky conditions. The system was placed on a platform, oriented toward the south with a 37° tilt, carrying, on the front surface, the PV module, the pyranometer and the temperature sensor. The PMS, SC and node were positioned underneath the platform to prevent excessive heating.

**Figure 9:**
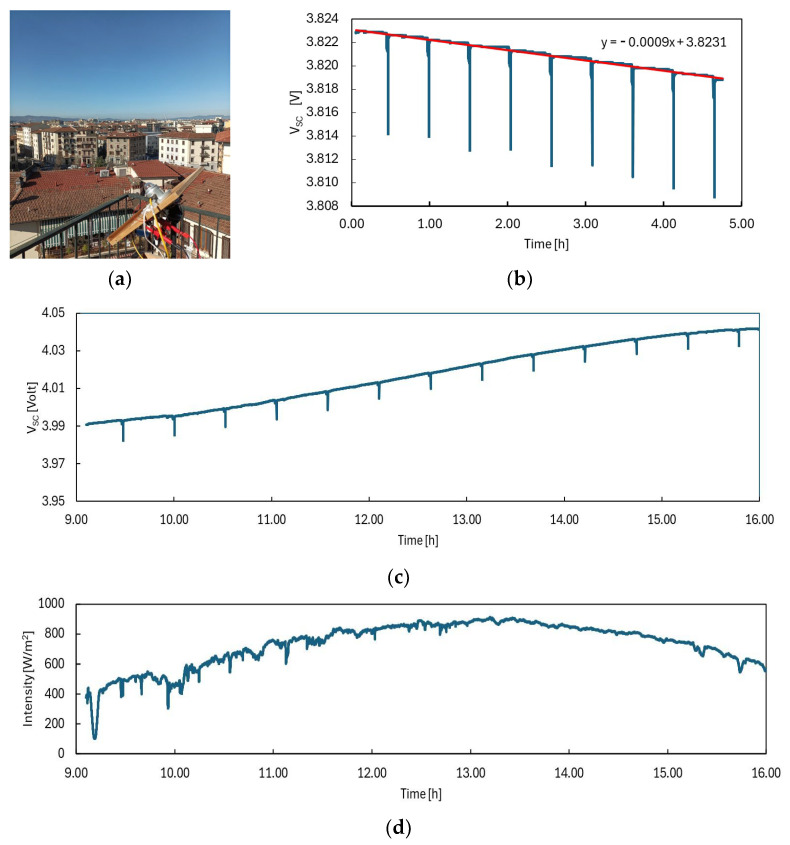
(**a**) Outdoor setup in Florence, Italy. Voltage across the SC during measurements (**b**) at night and (**c**) in daylight, with WSN transmission every 30 min, on a day with a clear sky, March 22, 2024. (**d**) Intensity measured on the same day by the pyranometer.

As an example, Figure 9b,c show the voltage measured across the SC vs. time with transmission every 30 min, respectively, during night- and daytime. Figure 9d shows the intensity measured by the pyranometer during the daylight measurement.

We observe that the voltage derivative as a function of time is proportional to the irradiation intensity, $\frac{dV}{dt}\propto \;\varphi$, as expected. When considering a 24 h day–night period, we measured a 35 mV total increase in voltage across the supercapacitor, corresponding to U_sc_ = 0.54 Wh energy stored in the supercapacitor.

## 4. Discussion

In the proper voltage range of the supercapacitor (3.6 V to 4.2 V), the energy stored in the SC is a linear function of the voltage across its electrodes, *V_sc_* (see Figure 6b). This is useful, as we can discuss the exchanged energies in terms of changes in *V_sc_* during operation, a parameter easy to measure directly. To discuss the change in *V_sc_* as a function of time, we consider three contributions: Δ*V_out_*, Δ*V_in_* and Δ*V_quiesc_*. The first, which is always negative, is associated with the node activity; the second, which is always positive, is due to the PV module supply under radiation; and the third is associated with the consumption needed to power up the PMS. In terms of incremental contributions per unit time, we define the following:

(1) $$G_{tot}=G_{out}+G_{in}+G_{quiesc}={\left(\frac{∆V}{∆t}\right)}_{out}+{\left(\frac{∆V}{∆t}\right)}_{in}+{\left(\frac{∆V}{∆t}\right)}_{quiesc}\;$$

**Figure 10:**
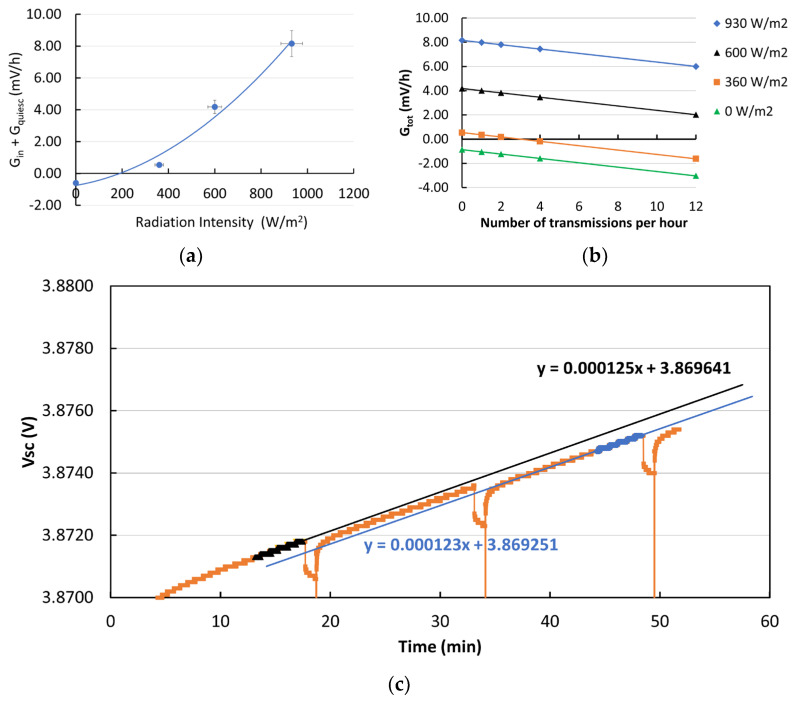
(**a**) Incremental contribution per unit time of the voltage across the supercapacitor, $G_{in}+G_{quiesc}$ plotted as a function of radiation intensity. The best fit is obtained with a second-order polynomial. (**b**) Total voltage change per unit hour, *G_tot_*, plotted as a function of the number of transmissions per unit hour for different radiation intensities. (**c**) A plot of the voltage across the supercapacitor, *V_sc_*, measured as a function of time under Sun Simulator illumination in the case of 350 W/m^2^ and node sensing/transmission every 15 min. The voltage drop due to two consecutive node transmissions is evidenced by the difference between the intercepts of the corresponding best-fit lines (see text).

From the measurements shown in Figure 8a, taken when the node is sleeping, we can determine $G_{quiesc}={\left(\frac{∆V}{∆t}\right)}_{quiesc}$ from the slope of the *V_sc_*–time curve in the case of null radiation intensity. We obtain $G_{quiesc}=0.01\;\frac{mV}{min}$. This corresponds to a power consumption of 9.3 mW and to an energy loss *U_quiesc_* = 0.14 Wh. From measurements at 350, 600 and 930 W/m^2^ light intensity (see Figure 8a), we can determine $G_{in}={\left(\frac{∆V}{∆t}\right)}_{in}$ from the slope of V_sc_(t) at different radiation intensities. As a result, we obtain $G_{in}+G_{quiesc}$ as a function of radiation intensity, as shown in Figure 10a (for error bar estimation, see Section 2). The best fit of this curve is obtained by exploiting a polynomial of the second order. Then, $G_{out}\;$can be determined by evaluating the voltage loss after each transmission at any radiation intensity applied. As an example, Figure 10c shows a plot of the voltage across the supercapacitor, *V_sc_*, measured as a function of time under irradiation equal to 350 W/m^2^ and with node wakeups every 15 min. The voltage drop due to two consecutive node wakeups is evidenced by the difference between the intercepts of the best-fit lines immediately after the transmission takes place. The loss of voltage due to node consumption is evaluated as the difference between the change in the intercept of the voltage–time line when the node is sleeping between two consecutive transmissions. We obtain a voltage drop for each wakeup equal to Δ*V_tr_* = −0.18 mV, a value independent of the irradiation intensity. In terms of the corresponding energy stored in the supercapacitor, we obtain *U_tr_* = 2.8 mWh. *G_tot_* is then determined as a function of the number of wakeups per unit time and of the radiation intensity, as shown in Figure 10b.

**Figure 11:**
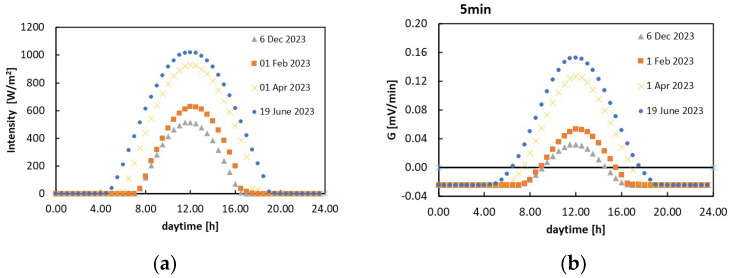
(**a**) The insolation curve measured on four days with a clear sky in 2023 [25] and (**b**) the corresponding voltage changes per unit minute, G, estimated in the case of transmission every 15 min.

From the plots in Figure 10, knowing the insolation curve during the day, it is possible to compute the corresponding change in voltage per unit time. As an example, Figure 11a,b show, respectively, the insolation curve measured on four days with a clear sky in 2023 (data from NREL database [25]) and the corresponding voltage change per minute in the case of transmission every 15 min calculated with this model.

**Figure 12:**
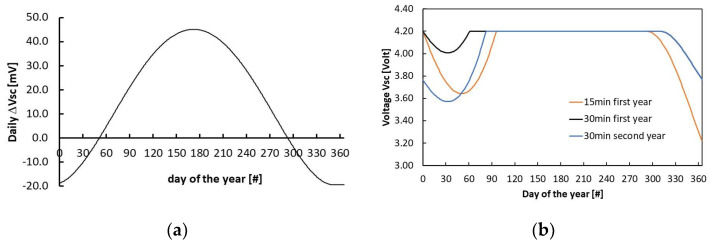
(**a**) The voltage change across the supercapacitor per day of the year, estimated in the case of transmission every 30 min. (**b**) The daily value of *V_sc_* estimated during the first year of operation for transmission every 15 min (red line) and 30 min (black line), starting from a full SoC (*V_sc_* = 4.2 V); the same calculation (blue line) in the case of the second-year operation for transmission every 30 min, starting from the SoC estimated at the end of the previous year (corresponding to *V_sc_* = 3.8 V).

By summing up the contributions throughout the day, we obtain the voltage change per day for that particular insolation curve: as an example, in Figure 12a, we show the voltage change calculated throughout the whole year in the case of transmission every 15 min. Figure 12b shows the voltage across the supercapacitor evaluated in the case of sensing/transmitting, respectively, every 15 min and 30 min during the first year, starting with a 100% state of charge of the battery, corresponding to 4.2 V. We observe that, in the case of wakeups every 15 min (red line), *V_sc_*, at the end of the first year, is below the proper range required for the supercapacitor (*V_sc_* as low as 3.2 V), indicating that, energetically, the system is unbalanced. On the contrary, transmission every 30 min shows a *V_sc_* decrease at the end of the year only down to 3.8 V. The same calculation carried out for the second year, where *V_sc_* starts from this as the initial value, shows that *V_sc_* is still within the required range (blue line). The decrease in *V_sc_* observed in the wintertime is balanced by its increase during the spring-to-autumn period, guaranteeing the self-sufficiency of the WSN for two whole years. Moreover, during successive years, *V_sc_* should repeat the trend of the second year, so we can expect *V_sc_* to remain within the proper range (3.6 V, 4.2 V) for virtually unlimited operation periods. 

This simple model was obtained from the outcomes achieved during the Sun Simulator tests. However, as we know that under solar radiation, the PV module is more efficient than under the Sun Simulator due to the better match of the triple-junction spectral response with the solar spectrum, even more promising results are expected for outdoor deployments. In fact, the total voltage change in the case of the complete night–day operation with a clear sky, as in Figure 9c, is estimated by the model to be equal to 32 mV, slightly lower than what was actually measured, that is, 35 mV.

**Figure 13:**
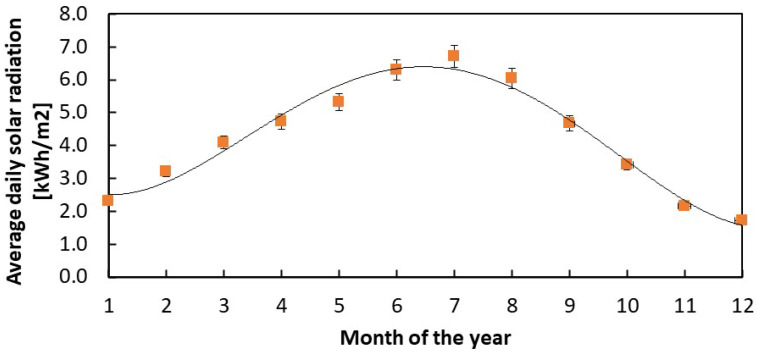
Monthly normal global average daily radiation measured in Siena, Italy, for the years from 2006 to 2022. Data from the ENEA Solaritaly database [26].

Before concluding, we need to mention here that the model discussed in this paper does not consider weather effects, such as partial insolation due to cloud coverage, and is based only on clear-sky insolation curves. To extend the discussion accordingly, a more detailed model should be used considering more realistic solar radiation intensity curves, directly measured at a specific location throughout the year and averaged over a significant number of years. In the case of Italy, these curves are available from a database by ENEA, Italy [26], as a listing of the average daily radiation intensities as a function of each month of the year, averaged for the years from 2006 to 2022 for several locations. As an example, the monthly curve of normal global average daily radiation for Siena, Italy, is shown in Figure 13. Error bars due to 5% uncertainty in the intensity are added to the plot (see Section 2).

In the case of March, we have ϕ_day_ = 4.1 kWh/m^2^. Considering our PV module, with an active area of 8.4 cm^2^ and 24% efficiency when connected to the PMS, the average energy released daily by the panel would be U_PVtheo_ = 0.86 Wh, a value that compares favorably to the one we can extract from the outdoor measurements reported in Figure 9c. In fact, our 24 h outdoor measurements made in March corresponded to U_sc_ = 0.54 Wh energy stored in the supercapacitor. As the power consumption of the system is 0.14 Wh and the energy due to the WSN activity throughout the whole day, in case of transmission every 30 min, is U_WSN_ = U_tr_ × 24 × 2 = 0.13 Wh, the total solar panel energy from the PV module can be calculated at U_PVexp_ = 0.81 Wh, a value in agreement with what is expected from the data shown in Figure 13. 

## 5. Conclusions

The development and testing of a novel prototype system for WSN ambient monitoring have been presented and discussed. The device, based on eight GaAs-technology triple-junction solar cells with an 8.4 cm^2^ total active area coupled with a 4000 F/4.2 V hybrid supercapacitor, proved to be able to self-power a WSN with 10 mW power consumption for a 1 min long operation and transmission every 30 min for virtually unlimited periods. Although the tested power consumption is compatible with that of new-generation LED-based NDIR sensors for CO_2_ measurement, these results can be extended to a wider range of sensors with similar or lower power demand, such as temperature and humidity sensors or electrochemical sensors for O_2_, CO, NO_x_ and VOC measurements.

The presented outcomes are aimed at providing a preliminary evaluation of the energy sustainability of the system. On the basis of these promising results, our future goal is the validation of the system through prolonged outdoor deployments to actually demonstrate the possibility of realizing energy-sustainable low-power systems relying on small-sized multi-junction PV panels and novel hybrid supercapacitors. The prototype is now under further development to exploit the first application with innovative low-consumption miniaturized NDIR LED-based CO_2_ sensors [8]. Moreover, further engineering is required to make the system compliant with applications in outdoor scenarios. To this end, the effect of external factors that could worsen the harvesting efficiency of the PV modules, e.g., dust and pollution causing exceptionally low radiation intensities with prolonged exposure, will be evaluated. Finally, on the basis of our promising results, an extended model including weather effects on insolation and the corresponding sizing of the PV/supercapacitor/PMS in view of the self-powering the miniaturized WSN are now under further development, and they will be the subject of forthcoming work.

## Figures and Tables

**Table 1:** Parameters of the supercapacitor C424000R used in this study [18].

Parameter	Measurement Unit	Nominal
Capacitance	F	4000
Voltage	V	4.2
Max recharging voltage	V	4.2
Energy storage	Wh	14
Internal resistance (AC)	mΩ	45
Normal current	A	2
Leakage current	mA/72 h	≤0.5
Cycle life	#	≥100,000
Operating temperature range	°C	−40 ÷ 65
Storage temperature range	°C	−40 ÷ 70

**Table 2:** Specifications of the power management system used in this study [20].

Parameter	Measurement Unit	Nominal
$V_{in}$	V	6 ÷ 24
$V_{charge\;max}$	V	4.2 ± 1%
$V_{discharge\;max}$	V	2.9 ± 1%
$I_{quiesc}$	mA	<2
T	°C	−40÷80
η	%	78

**Table 3:** Measured parameters of the triple-junction module under 1000 W/m^2^ AM1.5G.

Parameter	Measurement Units	Sun Simulator	Solar Irradiation
$I_{sc}$	mA	24.0	26.0
$V_{oc}$	V	10.10	9.88
$V_{max}$	V	9.09	8.74
$I_{max}$	mA	21.0	23.7
$V_{out}\;$	V	7.65	7.65
$I_{out}$	mA	22.8	24.9
FF	%	78.7	80.6
$P_{max}$	mW	191	207
$P_{out}$	mW	174	190
$\eta_{max}$	%	24	26
$\eta_{out}$	%	22	24
T	°C	35	41

## Data Availability

In the Discussion Section, this paper used solar radiation data from the NSRDB—National Solar Radiation Database—of the National Renewable Energy Laboratory (NREL), U.S. Department of Energy, Office of Energy Efficiency and Renewable Energy, Alliance for Sustainable Energy LLC, https://nsrdb.nrel.gov/, and from the ENEA Solaritaly Database, URL (accessed on 28 August 2024) Agenzia nazionale per le nuove tecnologie, l’energia e lo sviluppo economico sostenibile, dipartimento tecnologie energetiche e fonti rinnovabili C.R. ENEA Casaccia-Via Anguillarese 301, 00123 Roma (RM), Italy, http://www.solaritaly.enea.it/TabelleRad/TabelleRadIt.php. URL (accessed on 28 August 2024).

## Nomenclature

**Symbol**	**Parameter**	**Measurement Unit**
AM	Air Mass	
C	Capacitance	[F]
f	Radiant intensity	[W/m^2^]
FF	Fill factor	[%]
G	Incremental voltage rate across supercapacitor	[mV/min]
I	Current	[A]
I_max_	Current at peak power	[V]
I_out_	Current at PMS	[V]
I_sc_	Short-circuit current	[A]
P_max_	Maximum PV Power	[W]
η _max_	Efficiency	[%]
P_out_	Power at PMS	[W]
R_ESR_	Internal resistance	[W]
T	Temperature	[K]
t	Time	[s], [h]
U_PV_	Daily energy released by PV module	[Wh]
U_sc_	Energy stored in supercapacitor	[J]
U_WSN_	Energy required by WSN	[Wh]
V	Voltage	[V]
V_max_	Voltage at peak power	[V]
V_oc_	Open-circuit voltage	[V]
V_out_	Voltage at PMS	[V]
Vsc	Voltage across supercapacitor	[V]
